# Individual and team competencies in translational teams

**DOI:** 10.1017/cts.2020.551

**Published:** 2020-10-21

**Authors:** Gaetano R. Lotrecchiano, Deborah DiazGranados, Jennifer Sprecher, Wayne T. McCormack, Damayanthi Ranwala, Kevin Wooten, Daniel Lackland, Heather Billings, Allan R. Brasier

**Affiliations:** 1 Department of Clinical Research and Leadership, George Washington University School of Medicine and Health Sciences, Washington, DC, USA; 2 Wright Center for Clinical and Translational Research, School of Medicine, Virginia Commonwealth University, Richmond, VA, USA; 3 Institute of Translational Health Sciences, University of Washington, Seattle, WA, USA; 4 Department of Pathology, Immunology and Laboratory Medicine, Clinical and Translational Science Institute, University of Florida College of Medicine, Gainesville, FL, USA; 5 Academic Affairs Faculty, South Carolina Clinical and Translational Research Institute, Medical University of South Carolina, Charleston, SC, USA; 6 Department of Management, College of Business, University of Houston-Clear Lake, Houston, TX, USA; 7 Institute for Translational Sciences, University of Texas Medical Branch, Galveston, TX, USA; 8 Department of Neurology, Medical University of South Carolina (MUSC), Charleston, SC, USA; 9 Center for Clinical and Translational Science, Mayo Clinic, Rochester, MN, USA; 10 Institute of Clinical and Translational Research, School of Medicine and Public Health (SMPH), University of Wisconsin-Madison, Madison, WI, USA

**Keywords:** Translational science teams, team science, team science competencies, individual competencies, team competencies

## Abstract

Translational scientists create, advance, and translate knowledge as a result of research, learning, and application. Translational teams are composed of dynamic and diverse interprofessional and cross-disciplinary members that generate new knowledge to address a shared translational objective. The objective involves advancing an interventional product, behavioral intervention, or evidence-based approach to improve human health. This paper focuses on identifying individual and team competencies using a modified Delphi method to reach a consensus on the competencies needed by translational teams (TTs).

## Introduction

With the increasing emphasis on research programs to address complex health and societal problems, a grass-roots revolution in interprofessional and cross-disciplinary team approaches is occurring in the scientific community [1]. This research revolution is driven by a number of factors, including increasing depth of research disciplines, focus on real-world applications, enhanced productivity, and utilization of research projects [2]. An abundance of social science research from disciplines such as organizational psychology, social psychology, sociology, philosophy, leadership studies, and communications has focused on team effectiveness and can be used to inform our understanding of translational teams (TTs). With the increased emphasis on enhancing team outcomes, substantial effort has been invested in comprehensive reviews and meta-analyses that have resulted in the identification of competencies (i.e., knowledge, skills, abilities, and attitudes) that are needed to advance team performance [3–5].

Previous studies have indicated that appropriately applied team training substantially impacts team performance and innovation [6, 7]. In particular, training efforts focusing on knowledge, skills, and abilities that are content-appropriate can result in substantial transfer and positive outcomes [8]. It is important to consider the context in which teams function. TTs, typically located in academic institutions, operate in a complex organization that has unique characteristics and implications for training. Because these training activities are most impactful when tailored to the team context, a great need arises to identify evidence-informed competencies most relevant to TTs with the goal of enabling trainees to successfully participate in TTs, enhance the productivity of TTs that they participate in, and derive satisfaction from participating in research as teams.

A TT, in line with the formal definition of a team [9], is composed of diverse members who interact, adapt, and evolve using established norms and defined roles to address a shared translational objective. Diverse members involve multiple perspectives, professions, career stages, stakeholders (patients, communities), and other voices appropriate for its developmental stage. The objective of a TT involves advancing a product (device/drug/diagnostic), behavioral intervention, or evidence-based approach to sustainable improvements in human health. A TT may work in one or more phases of translation, including preclinical, clinical, implementation, and population-based research, as well as in the process of translation itself. Building on this definition of a TT, our work seeks to identify core competencies that would inform relevant evidence-informed training and evaluation. To accomplish this goal, a comprehensive literature review was conducted and interpreted by the authors of this manuscript, who are a group of CTSA Team Science experts, using a modified Delphi approach.

## Materials and Methods

A nine-member sub-group of the Team Science Affinity Group (TSAG) formed to address the glaring need to develop a taxonomy of competencies for TTs. TSAG is a community comprised of team science scholars, TT investigators (e.g., principal investigators advocating team science processes, members of scientific teams), evaluators, and educators who span the CTSA network, which meets monthly to share knowledge, generate best practices, collaborate on field studies, and provide advocacy for TT science. The nine members of the sub-group (representing nine distinct CTSA hubs) were subsequently charged by the CTSA Methods and Processes Domain Task Force to review existing team science models and prepare a set of competencies for the TT science at the individual and team level.

A modified Delphi process consisting of (1) a literature review, (2) an initial list of competencies, (3) successive rounds of review and analysis, and (4) iterative edits resulted in consensus among the authors for a definition of translational science teams and a competency framework inclusive of individual and team components [10, 11].

Individual competency-relevant literature was identified using the search strategy (“Translational Medical Research”[Mesh] OR “translational research” OR “translational science” OR “translational medical research” OR translational[tiab] OR transl[All Fields] OR (translational[tiab] AND (scientist* OR researcher*))) AND (team OR teams OR teamwork OR collaboration OR cooperative OR cooperation OR “Intersectoral Collaboration”[Mesh] OR “Interprofessional Relations”[Mesh]) AND (skill OR skills OR competency OR competencies OR competence OR “Professional Competence”[Mesh] OR “Competency-Based Education”[Mesh]) NOT (animals[mh] NOT (humans[mh] AND animals[mh])). Group competency-relevant literature was identified using the strategy: (“Translational Medical Research”[Mesh] OR “translational research”[tiab] OR “translational science”[tiab]OR “translational medical research”[tiab] OR translational[tiab] OR transl [All Fields]) AND (team OR teams OR teamwork OR “Intersectoral Collaboration”[Mesh] OR “Interprofessional Relations”[Mesh]) AND (“Cooperative Behavior”[Mesh] OR “Group Processes”[Mesh] OR “group processes” OR “group process” OR “group behavior” OR “group behaviors” OR “group behaviour” OR “group behaviours” OR “cooperative behavior” OR “cooperative process” OR “cooperative processes” OR “group processes” OR “group process” OR “team communication” OR “team mentoring”).

Competencies described in the literature review were identified, analyzed and categorized as individual, team, and/or organization. This iterative work was conducted during monthly and bi-monthly virtual meetings that afforded an opportunity to promote understanding and generate consensus. The consensus was defined a priori as ≥80% of the nine members supporting the iterations, and subsequent versions. When consensus was lacking members sought out substantiating literature to inform final decisions. An online information management tool (Trello) provided a forum for shared decision making, a resource repository, and a communications archive. A video conferencing tool (Zoom) was used to host the meetings, and no anonymous polling or voting tools were used.

The first outcome of the modified Delphi was consensus that the competency category of *organization* was out of scope for this project and would be explored through future studies. The second outcome was a working definition of translational science teams and a draft of competencies that were presented at the June 2019 Science-of-Team-Science conference at Michigan State University. Conference attendees provided feedback and expert opinion was solicited through targeted inquiry. Additional input from experts in the field of team science was captured during question and answer sessions following formal presentations to the TSAG large group monthly meetings and the Methods and Processes Domain Task Force. This qualitative data guided the next stage of the project, which was to finalize definitions, tag each competency as individual or team, and map each competency by primary and secondary strength of association to one or more of the five domains outlined in Table 1. Table 1 depicts the definition of each of these five domains, as well as an example of how they might be demonstrated in translational science. This stage followed a similarly modified Delphi process of (1) proposed organization, (2) successive review and discussion, (3) iterative edits, and culminating in (4) group consensus on subsequent version(s). Consensus for a definition of translational science teams, and a list of 13 competencies mapped to 1 or more of the within 5 domains, was achieved after 4 rounds of review and non-anonymous voting. The mapping of competencies to domains included a primary association which noted the highest level of association and secondary association(s) which noted a lesser, but significant domain association that followed the same Delphi process indicated above.

**Table 1.** Competency domains and definitions

Domain	Definition and examples
Facilitating team affect [12]	Definition: Emotional bonds between team members that are grounded in expressions of genuine care and concern for the welfare of others including empathy, affiliation, and rapport on the basis of shared regard for the others Example: Expression of appreciation between team members involving sacrifices made to meet publication or grant deadlines regardless of the personal toll taken upon individual members
Team communication [13]	Definition: The skill to exchange and integrate knowledge and expertise through interpersonal, relational, organizational, and pedagogical means Example: Team member comments or restatement of translational research projects goals which combine the ideas of patients, leading scientists, and policy makers regarding the long-term plans for new standard of care
Managing team research [14]	Definition: Managing research and development organizations is, to a great degree, the art of integrating the efforts of its many participants Example: Specific actions taken to organize, plan, and execute components of a multi-institutional research study so that regulatory barriers for approval are addressed and an application is approved in a timely fashion
Collaborative problem solving [15, 16]	Definition: CPS is the cognitive and social skills allowing teams to integrate group achievements with team members’ idiosyncratic knowledge Example: Symbolic and behavioral actions on the part of investigators to connect the motives of team members to the higher order of developing a preventative protocol for a deadly disease prior to onset, and thus creating a shared vision to address from numerous disciplinary perspectives
Team leadership [17]	Definition: The cognitive, motivational, affective, and coordination processes associated with influencing organizational team performance Example: Comments made by a Principal Investigator or a senior team member that might encourage team members to think more broadly about the research question, or truly consider perspectives provided by team members representing diverse disciplines

## Results

The review of the literature informed a five-domain construct in which to organize by primary and secondary association five individual and eight team-level competencies specific to TT (Table 2). Primary and secondary association to a domain can be used by teams to focus on the identification of competency-based challenges by starting at the domain level and then considering the primary and then secondary competencies that are gaps at the team or individual level.

**Table 2.** Individual and team competencies organized by domain

Translational team competencies	Competency domains				
	Facilitating team affect (bonding)	Team communication	Managing team research	Collaborative problem solving	Team leadership
Individual competencies					
Facilitating awareness and exchange	++	+			++
Cognitive openness and intersubjectivity	+			++	++
Self-awareness	+	++		++	++
Interdisciplinary research management			+		++
Passion and perseverance	++				+
Team competencies					
Team roles					+
Team-based communication	++	+			
Shared visioning			++		+
Understanding complexity		++		++	+
Team learning and adaptive behaviors	++	++	++	+	++
Meeting management			++		+
Interdisciplinary collaboration		++		+	++
Building trust	+	++			

Competencies are categorized by primary (**+**) and secondary (++).

**Fig. 1.**
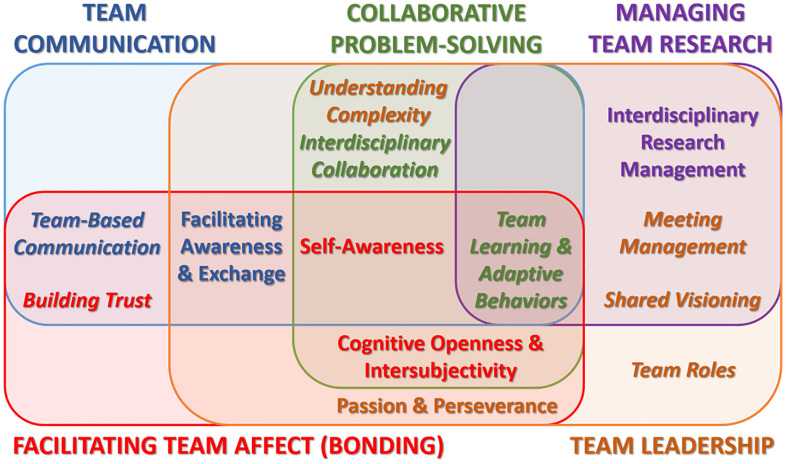
Overlapping and intersecting competencies across the domains. Colors define the primary domain for each competency.

We found that a critical dynamic to all domains is trustworthiness. It serves as a multifaceted dimension that is assumed in all domains grounded in ability, benevolence, and integrity. This is evidenced in each domain on both the individual and team levels [18]. These dimensions contribute to an environment of psychological safety that is uniformly accepted as a key component in high-functioning science teams [19]. The psychological safety that trustworthiness affords within a team setting has been evidenced to create stronger ties between leaders and followers. This has been illustrated as a critical component to the increased functioning in teams as a foundation of elements of multiple dimensions including cooperation, coaching, communication, cognition, conflict, and coordination which serves as a mediator in developing cultures that sustain team success [8, 20, 21]. Its role in informing individual and team-level competencies comes from its definition as a psychological state consisting of a willingness to accept vulnerability and change to meet the positive expectations (trustworthiness) of others [18]. Fig. 1 defines the construct and the overlapping and intersecting competencies represent this focus on trustworthiness in teams. The five competency domains are labeled in capital letters, individual competencies are shown in regular font, and team competencies are shown in italics.

## Discussion

The development and implementation of structured competencies represent a timely and essential component of clinical and translational research. As the concepts of team science have been incorporated into clinical and translational research, specific competencies are needed to build and enhance training and workforce development. Though not within the scope of this paper, competency-based learning and workforce development in translational science will need to adopt common competencies if we expect that programs of study and ongoing career development can be designed and implemented. This is not to say that competency-based principles can always be integrated into our commonly employed techniques for training and learning. In fact, the success of competency-based learning is grounded in the ability to observe competence in multiple ways focusing on the behaviors, cognitions, attitudes, practices, etc. that evidence competence. It is, therefore, important that these competencies serve as a guide to the task of intervention development that ensures that science teams have access to systematic development through learning, exercise, and reflection. However, we believe that the competency association assists in the creation and organization of educational materials and the subsequent sharing of materials across the country.

The successful adoption of the competencies can effectively support the development of the next generation of scientists with skills for TT research. Individual and team competencies unique to team science are proposed to enhance the team research dynamics as an enduring practice and attitude to be experienced through all career phases and stakeholders. The successful implementation of these competencies will require the acceptance and commitment of researchers and institutional leadership. Institutional resources and policies should recognize team science as an essential cross-disciplinary and interprofessional base.

This study advances the development of translational science as a rigorous discipline in two important ways: (1) we define the distinguishing characteristics of a TT and (2) we identify the most appropriate competencies for TT training and evaluation. Because of the lack of consensus on what is meant by team science throughout the CTSA consortium, this study was vetted by the CTSA Methods and Processes Domain Task Force to advance a consensus definition of the TT. To reiterate, the TT incorporates a cross-disciplinary approach characteristic of an academic knowledge-generating team with the drug/device/intervention development characteristic of an industry product development team [22].

Tailoring professional development to the specific context substantially impacts team performance and innovation [23]. Previous work in the CTSA consortium by the Education Key Function Committee identified over 99 competencies for a translational scientist (https://clic-ctsa.org/education/core-competencies-clinical-and-translational-research). Consequently, team science-focused training approaches have been heterogeneous and difficult to evaluate. Using a modified Delphi method, our study derives from the larger body of literature in team science competencies to a subset of those we believe are most relevant to TT performance. A commentary co-authored by the NCATS Director articulated the need to more rigorously define the characteristics of a translational scientist that proposed high-level domains, many unique to the translational space [24]. This effort is the first step to close the gap between what educators and evaluators believe should occur in TTs, and what typically is observed in TTs. This effort should also include areas such as boundary spanning, systems-level thinking, and deep understanding of regulatory processes that would promote the advancement of a translational product and is not a characteristic of a traditional academic knowledge-generating team.

Much of the competency work has focused primarily on the knowledge, skills, and abilities of the individual and not of the team. Our analysis includes the definition of group-related competencies, such as team trust, which is not a property of an individual, but the collective result of interactions of individuals within the team context. Similarly, collaborative problem solving would be an emergent team property. These differentiating characteristics indicate the TT is a special case of the cross-disciplinary team and inform relevant competency domains for training to enhance its conduct and characteristics for evaluation. Our study serves as a foundation for designing professional development activities and for evaluating their impact on team performance, innovation, member satisfaction, and impact on health or health care delivery. To accomplish this goal, a comprehensive literature review was conducted and interpreted by the authors of this manuscript, who are a group of CTSA Team Science experts, using a modified Delphi approach.

## Limitations

The general study of team science competencies includes a wide array of competency as related to teaming in multiple science sectors. This paper focuses on TTs mainly in the context of clinical and translational science as understood through the lens of biomedical and health science teams. This work does not fully represent the broader discourse on team science competency.

## Conclusions and Future Research

We have built on previous work about team science competencies to focus specifically on the individual and team competencies required for successful TTs. The results of this work take into account the goals of health science professionals and their emphasis on interventional products, behavioral interventions, and evidence-based approaches that improve human health.

TTs are challenging operating environments in that they are practically based, inherently problem solving, and often cross-disciplinary in character. While it can be argued that a number of the competencies illustrated in Table 2 are applicable to many team settings, they have been socially constructed from expert opinion, and judged to have practical relevance to translational science. The findings from this study are based on the insights of subject matter experts, thus they need next to be investigated across the translational science community to establish this context-specific validity (i.e., translational science).

A potential implication of this study involves the intersection of competencies at both the individual and team levels which may largely contribute to the development of trustworthiness. Trustworthiness is considered key in multidisciplinary and scientific environments, due to the nature of the scientific enterprise and the interdependence and coordination needs in the production of knowledge. The implications of this study suggest the need for the empirical analysis of the multiplicative effects of numerous cognitions, skills, and abilities to manifest higher-order conditions such as trustworthiness. Exploration of such efforts would well serve and inform curriculum development for translational science, as well as for the development of existing TTs.

Application of these findings over the long term should focus on the identification of observable strategies that can support evaluation of these competencies through both individual and team led interventions. These evaluative efforts will need to address multidimensional assessment factors (affective, cognitive, behavioral, etc.) so as to clarify how multiple competencies can be observed and assessed in diverse teams. This strategy will lead to outcome research that can begin to inform how team science competence can be more fully developed, maintained, and applied to the development of novice through senior scientists, and other stakeholders.
